# A novel rat model of liver regeneration: possible role of cytokine induced neutrophil chemoattractant-1 in augmented liver regeneration

**DOI:** 10.1186/s13022-015-0020-3

**Published:** 2015-11-02

**Authors:** Dipok Kumar Dhar, Goran Hamid Mohammad, Soumil Vyas, Dieter Clemens Broering, Massimo Malago

**Affiliations:** Institute for Liver and Digestive Health, Royal Free Hospital, University College London, Pond Street, London, NW3 2PF UK; Department of Surgery and Interventional Sciences, Royal Free Hospital, University College London, Pond Street, London, NW3 2PF UK; Chemistry Department, School of Science, University of Sulaimani, Sulaimanyah, Kurdistan Region, Iraq; Department of Organ Transplantation Centre and Comparative Medicine Department, King Faisal Specialist Hospital and Research Centre, MBC 03 P.O. Box 3354, Riyadh, 11211 Saudi Arabia

**Keywords:** ALPPS, Portal vein ligation, Portal vein embolization, Cytokines, Cell proliferation

## Abstract

**Background:**

Liver resection is the mainstay of treatment for most of the liver tumors. Liver has a unique capability to restore the lost volume following resection, however, most of the primary tumors grow in a liver with preexisting parenchymal diseases and secondary tumors often present in multiple liver lobes precluding a safe curative resection. Two-stage hepatectomy and portal vein ligation (PVL) are used to achieve a safer future remnant liver volume (FRLV), however, these procedures take several weeks to achieve adequate FRLV. A recently introduced faster alternative two-stage hepatectomy, also know as associated liver partitioning and portal vein ligation for staged hepatectomy (ALPPS), produces a desirable FRLV in days.

**Methods:**

To have an insight into the mechanism of ALPPS associated liver regeneration, we reproduced a rat model of ALPPS and compared the results with the PVL group.

**Results:**

Our results convincingly showed an advantage of the ALPPS procedure over PVL group in terms of early regeneration, however, in 1-week time the amount of regeneration was comparable. An early regeneration in the ALPPS group coincided with an early entry of hepatocytes into the cell proliferation phase, a significant increase in portal pressure and increase in hepatic enzymes in the ALPPS group compared with the PVL group. According to the protein array evaluation of 29 cytokines/chemokines, cytokine induced neutrophil chemoattractant-1 had the highest expression whereas IL-6 had the highest fold (>6 vs PVL group) expression at the early phase of regeneration in the ALPPS group.

**Conclusions:**

This unique rat model of ALPPS would help to improve our understanding about the liver generation process and also will help in further refinement of the ALPPS procedure for the clinical benefit.

**Electronic supplementary material:**

The online version of this article (doi:10.1186/s13022-015-0020-3) contains supplementary material, which is available to authorized users.

## Background

With the advent of improved perioperative management, liver resection is getting a newer dimension with increasing number of extended hepatic resections at low morbidity and mortality rates. According to the current guidelines, approximately 75 % of the liver can be removed safely without increasing the risk of post-operative liver failure in patients with healthy liver, however, this decreases when the remnant liver is not healthy [1]. This rule could be applied to patients presenting with a well demarcated tumor(s) confined in a specified anatomical zone and/or without any preexisting parenchymal liver diseases. However, most of the patients with primary liver tumor already have some sort of preexisting liver parenchymal diseases such as cirrhosis and those with secondary tumors, often present with tumors scattered over multiple liver lobes precluding a straight forward line of resection and preservation of an adequate future remnant liver volume (FRLV). Excessive removal of liver parenchyma or an inadequate FRLV often culminates in postoperative morbidity and even death from liver failure [2].

Unlike other vital organs in the body, only liver sieves the gut originated portal flow before draining it out into the systemic circulation pointing towards a vital role of portal blood flow in sustaining normal liver function and growth. It has been well recognized that diverting portal blood flow by placing a portacaval shunt below the liver causes liver atophy [3]. In 1986, Kinoshita et al. first exploited this concept in humans and was successfully able to induce atrophy of the tumor bearing side of the liver by portal vein embolization with simultaneous compensatory hypertrophy of the FRL [4]. This paradigm has reined the strategy to increase the preoperative FRL volume for the last two decades with mixed outcomes. The main drawback of this approach, however, has been the time required to achieve an adequate increase of the FRLV. Usually it takes 3–12 weeks to achieve adequate FRLV following PVE, which often proved too long due to the unrestricted tumor growth [5]. This has prompted the surgeons to rethink about some alternative strategies including the staged hepatectomies. Very recently, Schnitzbauer et al. [6] has pioneered a new strategy of two-staged approach to achieve faster growth of FRL by combining the portal vein ligation and parenchymal partitioning between the ligated and non-ligated part of the liver which ideally prevents cross collateral flow between the lobes and increases the pace of the growth of the FRL. This procedure was later acronymed as associated liver partitioning and portal vein ligation for staged hepatectomy (ALPPS). The ALPPS procedure is still in it’s evolving stage and although it significantly improves the pace of the growth of the FRLV, however, preliminary reports indicate that it comes at a cost of increased postoperative morbidity and mortality justifying further improvement or modification of the technique [7, 8]. Therefore, it is worth to reproduce the ALPPS in an animal model to study the actual mechanism of the accelerated regeneration, refine the procedure and find ways to further improve it. To that end, we herein describe for the first time an animal model of ALPPS in rats and evaluated possible mechanism(s) of enhanced liver regeneration.

## Methods

### Animals

Male Sprague–Dawley rats, 6–8 weeks old and weighing between 200 and 250 g, were obtained from the Charles-River Laboratories UK Ltd. The animals were housed in a temperature controlled room (23 ± 2 °C), with a relative humidity of 50 ± 10 % and alternate light and dark conditions. They were given standard laboratory rodent chow. All animal experiments were conducted according to the Home Office guidelines under the UK Animals and Scientific Procedures Act 1986.

## Experimental group

The experiment was conducted in three groups of animals; (1) Sham group: abdomen was opened and closed after manipulation of the liver hilum, (2) PVL group: selective portal vein ligation of all branches except the branch to the right median lobe was done and (3) ALPPS group: selective portal vein ligation and liver parenchymal partitioning were done. Each group consisted of 6 rats and there were four time points including 24 h, 48 h, 96 h and 1 week.

### Surgical operation

**Fig. 1:**
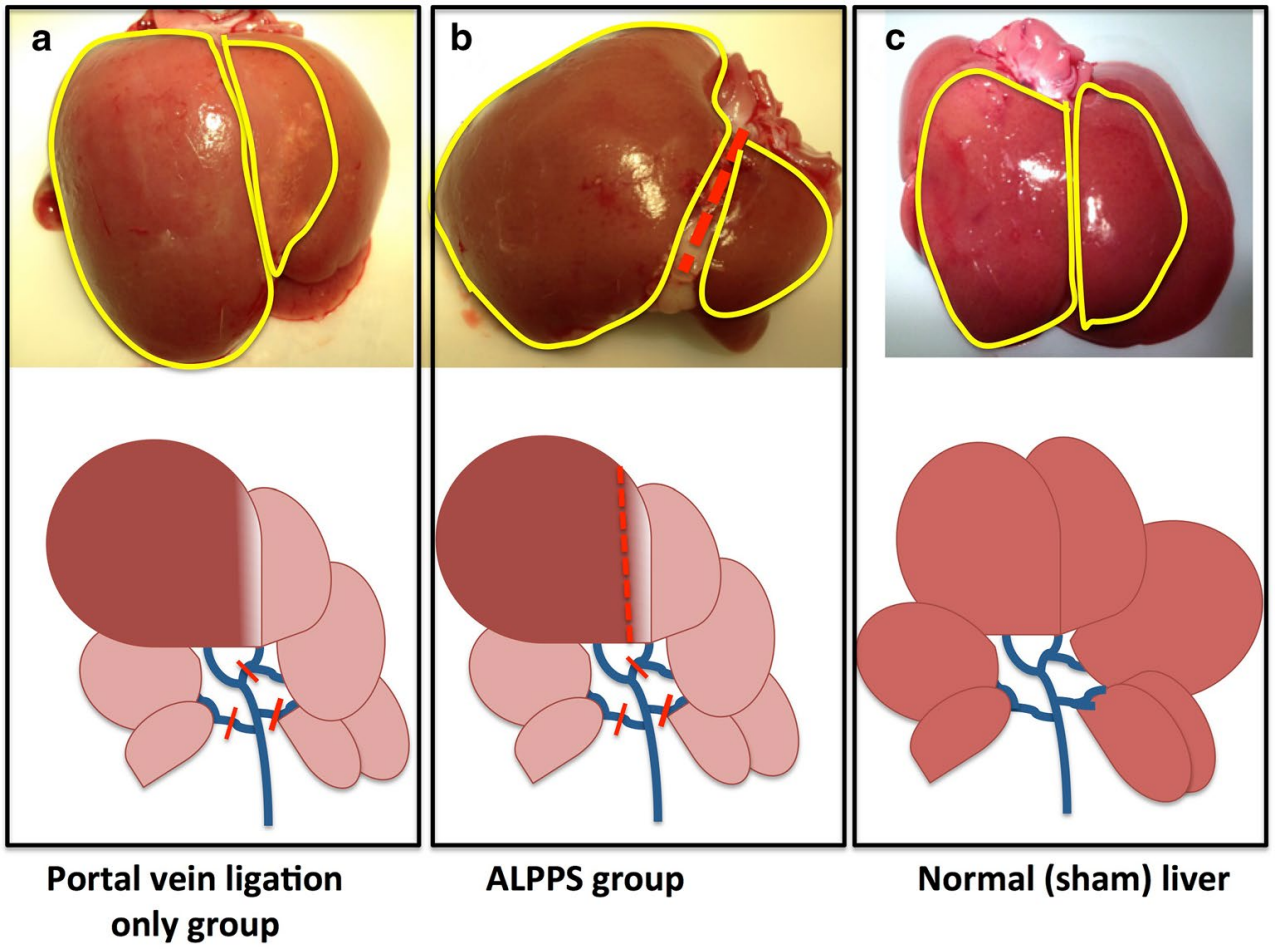
Experimental schema is shown. *Upper panel* is showing the liver at 1-week time point in different groups(**a** PVL, **b** ALPPS, and **c** Sham); the median lobes are encircled with *yellow line*. The *lower panel* shows the ligated portal branches (*red line*) and the parenchymal transection *line* is shown in *red* *interrupted line* in the ALPPS group (**b**)

Following the preoperative preparation, the skin was cleaned with iodine and 70 % alcohol. 2 % isoflurane with oxygen was used to induce anesthesia. Abdomen was opened through a midline incision and all hepatic ligaments were severed to free the liver. With the help of an operating microscope (Leica Wild M691, Wetzlar, Germany), hepatic hilum was inspected and all blood vessels including the portal branches and arteries were identified. Individual portal branches including the right portal vein, caudate branch, left median branch and the left lateral portal vein were dissected free from the arteries and were ligated near the pedicle with 7-0 nylon sutures (Fig. 1a, b). In the ALPPS group, in addition to the portal vein ligation the median lobe was transected along the vascular demarcation line visible following the PVL, which was immediately right to the median fissure of the median liver lobe (Fig. 1b). Parenchymal transection was achieved by ligating all vessels in the transection line with the help of 4-0 vicryl sutures and hemostasis was achieved by using hemoclips and diathermy cauterization. A bulldog clamp was applied briefly (3–4 min) near the hepatic pedicle (Pringle maneuver) to reduce the amount of blood loss during the parenchymal transection. A similar Pringle maneuver was also applied in the Sham and PVL groups.

All animals received 0.5 ml intraperitoneal normal saline before closure of the abdomen. Animals were allowed to recover in a heated (35 °C) chamber until they regained their consciousness and then were kept in a recovery room with heat pad under the cages for 24 h after the surgical operation. Body weight of all animals was monitored regularly. After 24 h, 48 h, 96 h, and 1 week time, animals were humanely killed. Immediately before killing, portal pressure was measured by inserting a 25G needle directly into the main trunk of the portal vein which was connected to a transducer pressure monitoring system [Powerlab (4SP) and Chart v5.0.1 software, ADInstruments Pty.Ltd, UK]. The pressure reading was taken in stable hemodynamic condition and for each reading the pressure was recorded over 1 min and the average was taken as the final pressure reading. Blood samples were collected from the abdominal aorta and the whole liver was dissected out for measurement of total liver weight and then individual lobes were dissected and weighed separately. Liver tissue samples were preserved in liquid nitrogen and also in 10 % buffered formalin for further use.

### Proteome profiler rat liver tissue cytokine array

We used a cytokine array system (Proteome Profiler Rat Cytokine Array; ARY008, R&D Systems, Minneapolis, MN, USA) to study the expression of multiple cytokines in liver tissue samples collected at different time points following the surgery. Briefly, 600 μg of pooled liver tissue homogenate collected from the same group was incubated with the array antibody cocktail provided with the kit and incubated for 1 h at room temperature before adding the sample to the array membrane preincubated with the blocking buffer and incubation overnight at 4 °C. Subsequently, the array membrane was incubated with the Streptavidin-HRP antibody for 30 min at room temperature and chemiluminescent detection reagent mix for 1 min. In between steps, the membrane was washed three times for 10 min each. The chemiluminescent signal was read by a image grabber (FluorChem M system, ProteinSimple, Santa Clara, California) and the data were analyzed with the MyImage analysis software (Thermo Fisher Scientific, USA). The pixel data were subtracted from the background noise and normalized by the reference values.

### Blood biochemistry

Plasma samples were used to measure the hepatic enzymes and liver function tests by using an automated clinical chemical analyzer (Roche P Module analyzer). All reagents and calibrators were supplied by Roche Diagnostics Limited, Charles Avenue, Burgess Hill, West Sussex, RH 15 9RY, UK.

### Routine histopathology and Immunohistochemistry

Paraffin embedded tissue sections were used for the hematoxylin and eosin staining. Immunostaining was performed by using a commercially available kit (Vector Laboratories, UK). Briefly, the slides were dewaxed in xylene, rehydrated in graded alcohol and rinsed in phosphate buffer solution (PBS) for 5 min. Antigen retrieval was done by immersing the slides in citrate buffer solution (pH 6.0, Dako, UK) and autoclaved for 15 min. Endogenous peroxidase activity was quenched by incubating the slides with 3 % hydrogen peroxide for 20 min. Following incubation with the blocking serum, the sections were incubated with anti-Ki-67primary antibody (monoclonal mouse anti-ki-67 antibody; Abcam, UK) overnight at 4 °C. Goat anti-mouse secondary antibody conjugated with IgG-HRP was applied for 30 min. Color development was done with the peroxidase substrate 3-amino-9-ethylcarbazole. Between steps, slides were rinsed with PBS. Counterstaining of nucleus was done with Mayer’s hematoxylin solution.

### Statistical analyses

All data are reported as mean ± SEM. Differences between groups were compared by unpaired t test or Mann–Whitney U test as appropriate. For comparison of multiple groups, one-way ANOVA with Turkey’s HSD posthoc test was used. Statistical significance was taken as p < 0.05 by SPSS software, version 22.

## Results

All animals survived the procedure and had smooth recovery during the postoperative period. However, as shown in the Additional file 1: Figure 1, during our pilot study, we occasionally noticed necrotic changes along the partitioning line in the liver and also in the left median lobe (LML), which represents a similar occurrence in patients following ALPPS operation. The whole liver weight/body weight ratio was marginally higher in the ALPPS group when compared with the PVL and the Sham groups, however, there was no statistical differences among the groups (data not shown).

**Fig. 2:**
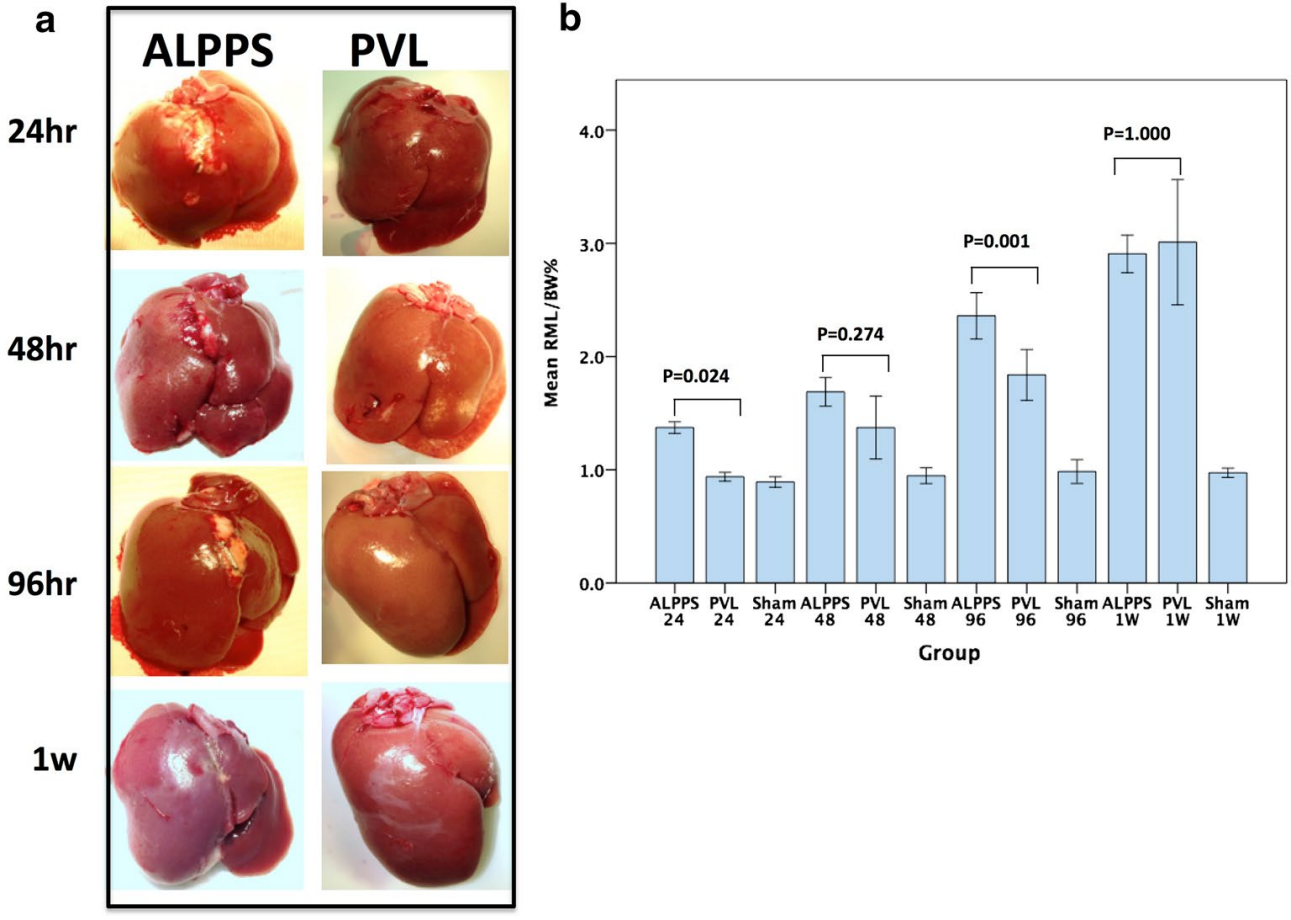
The *left panel* (**a**) is showing the macroscopic views of the explanted livers at different time points in the ALPPS and the PVL group. The *right panel* (**b**) is showing the ratio of the FRL (*right* median lobe)/body weight (FRL/BW) ratio. Significant increases in the FRL/BW ratio was noticed at 24 and 96 h time points in the ALPPS group compared with the PVL group

There were steady and significant increases in the FRLV over the time period in both ALPPS and PVL groups. Compared with the Sham livers, the ratio of FRLV/BW increased 54.0, 78.2, 139.8 and 198.9 % in the ALPPS group and, 5.1, 44.9, 86.8 and 209.4 % in the PVL group at 24 h, 48 h, 96 h and 1 W, respectively (Fig. 2a, b). When compared with the PVL group, significant increases in the FRLV/BW ratio were noticed in the ALPPS group at 24 h (p = 0.024, ANOVA/Turkey HSD) and 96 h (p = 0.001, ANOVA/Turkey HSD) time periods and it was almost similar in both groups at 1 week time period (Fig. 2). A contemporary atrophy of the ligated lobes including the LML was more pronounced in the PVL group than in the ALPPS group (data not shown).

**Fig. 3:**
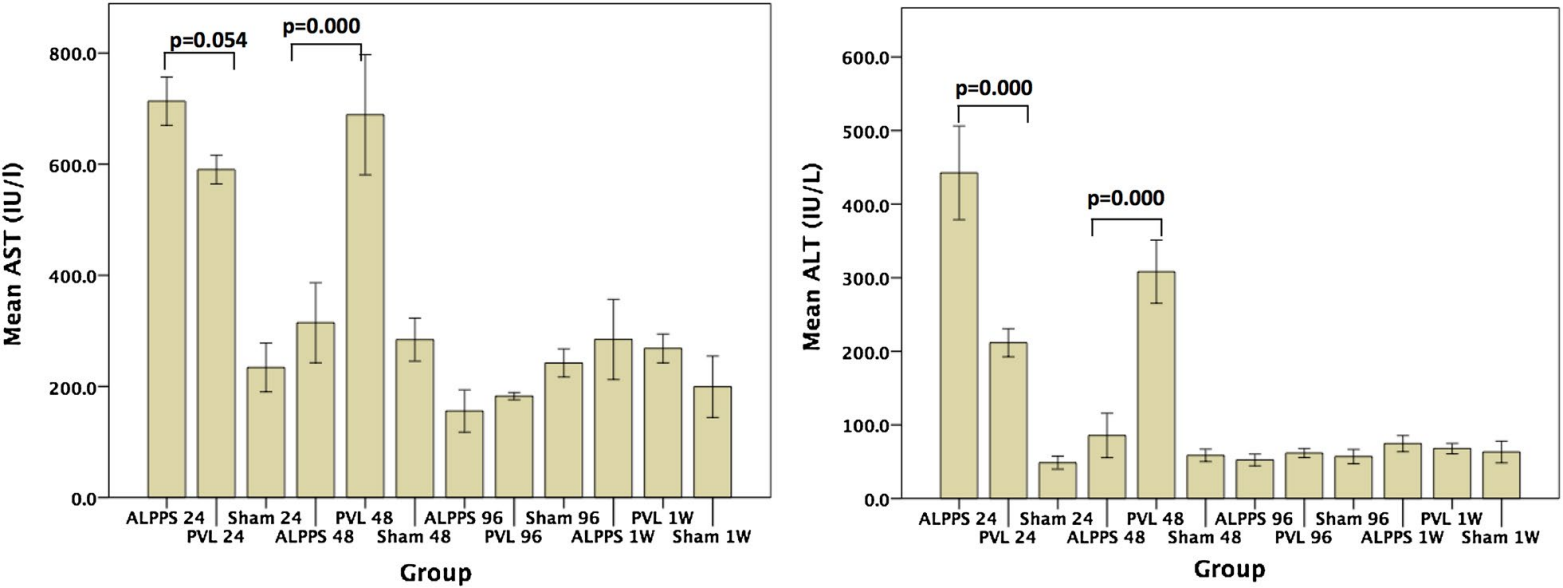
Hepatic enzyme (AST & ALT) levels are shown. Both enzymes had peak at 24 h time point in the ALPPS group whereas the peaks were at 48 h in the PVL group

Early peak levels in both serum aspartate aminotransferase (AST) and Alanine aminotransferase (ALT) levels were noticed at 24 h in the ALPPS group (AST, p = 0.054 and ALT, p = 0.000 vs. PVL group ANOVA/Turkey HSD test) whereas in the PVL group, the peaks were at 48 h for both enzymes (AST, p = 0.000 and ALT, p = 0.000, ANOVA/Turkey HSD test) (Fig. 3). Both enzymes retuned to almost normal level at 96 h postoperatively.

**Fig. 4:**
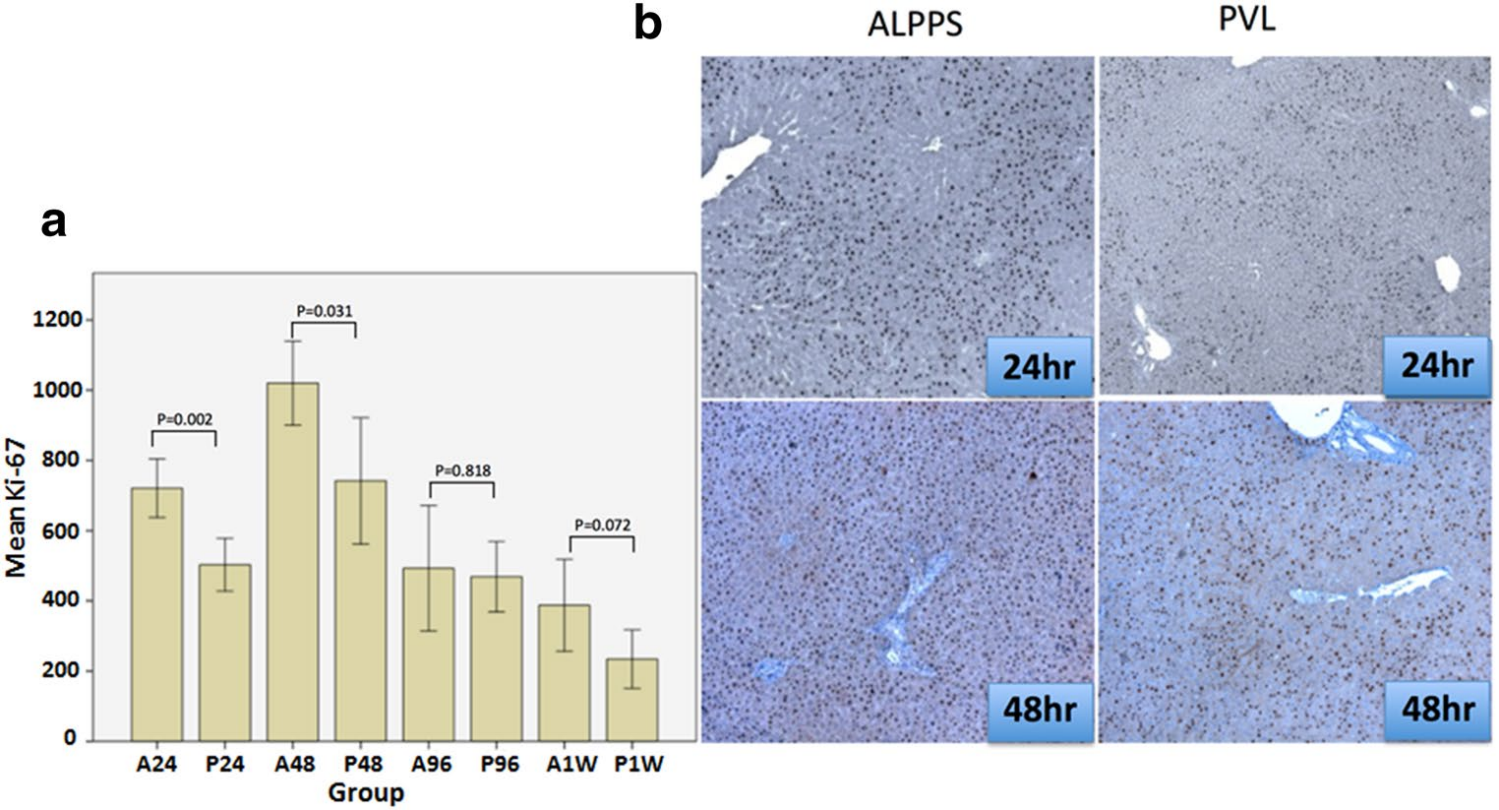
The proliferative index (PI) is shown in *panel* **a**, significant increase in PI was noticed at 24 and 48 h time points in the ALPPS group when compared with the PVL group. *Panel* **b** shows the immenohistochemical staining of Ki-67 in the ALPPS and the PVL group, Ki-67 stained cells were noticed near the portal tract area in the early time point (24 h) and dispersed all over the hepatic lobes at 48 h time point (×40)

**Fig. 5:**
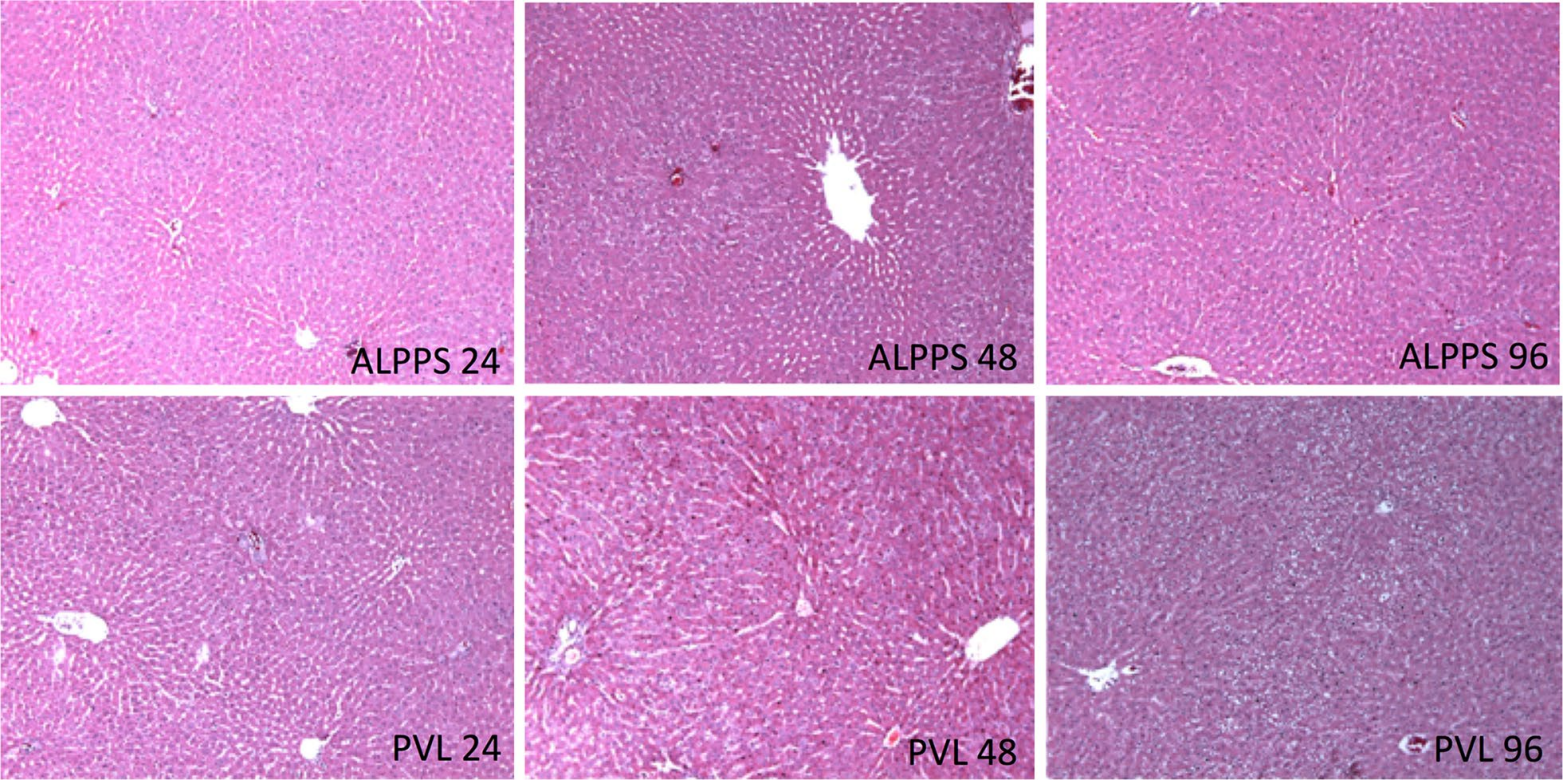
Hematoxylin and eosin stained tissue sections are shown. There was significantly higher infiltration of inflammatory cells in the ALPPS group at early hours (24 and 48 h) when compared with the PVL group (×40)

Next, we looked at the proliferative index (PI) of the liver at different time intervals as assessed by counting the number of Ki-67 positive nuclei per high power field (×100). Two individual authors who were blinded about the groupings did the counting and an average was considered as the final count. The number of cells in three high power fields (×100) were counted and the data was expressed as PI. Increased PI was noticed in the ALPPS group when compared with the PVL group throughout the experimental period and was significant only at 24 h (p = 0.002, t test) and 48 h (p = 0.031, t test) time points (Fig. 4). The inflammatory cells were counted in three high power fields(×100) in mostly populated areas and an average was considered as the final count for each section. The histopathological examination of H&E stained sections showed higher infiltration of liver by inflammatory cells in the ALPPS group at initial time periods (ALPPS vs PVL: 68 ± 23 and 79 ± 43 vs 37 ± 19 58 ± 36 at 24 & 48 h, p = 0.021 and 0.380, respectively) when compared with the PVL group, which was partially resolved at later time periods (Fig. 5). There was heterogeneity in the extent of infiltration with more infiltration closer to the line to partitioning and less away from the line.

The average portal pressure (PP) in the Sham group was 8.48 ± 1.19, 8.76 ± 0.95, 7.89 ± 0.37 and 8.14 ± 0.58 mmHg, at 24 h, 48 h, 96 h and 1 W, respectively (Additional file 2: Figure 2). Overall, the PP was highest in the ALPPS group at all time points except at 96 h and it was significantly higher in the ALPPS group only at 24 h when compared with the PVL group (ALPPS vs. PVL: 12.90 ± 1.32 vs. 10.04 ± 2.00, p = 0.025, 11.82 ± 2.11 vs. 10.60 ± 1.04, p = 0.210, 10.44 ± 2.05 vs. 10.76 ± 1.89, p = 0.766 and 12.03 ± 1.52 vs. 9.54 ± 2.06, p = 0.129, at 24 h, 48 h, 96 h and 1 W, respectively).

**Fig. 6:**
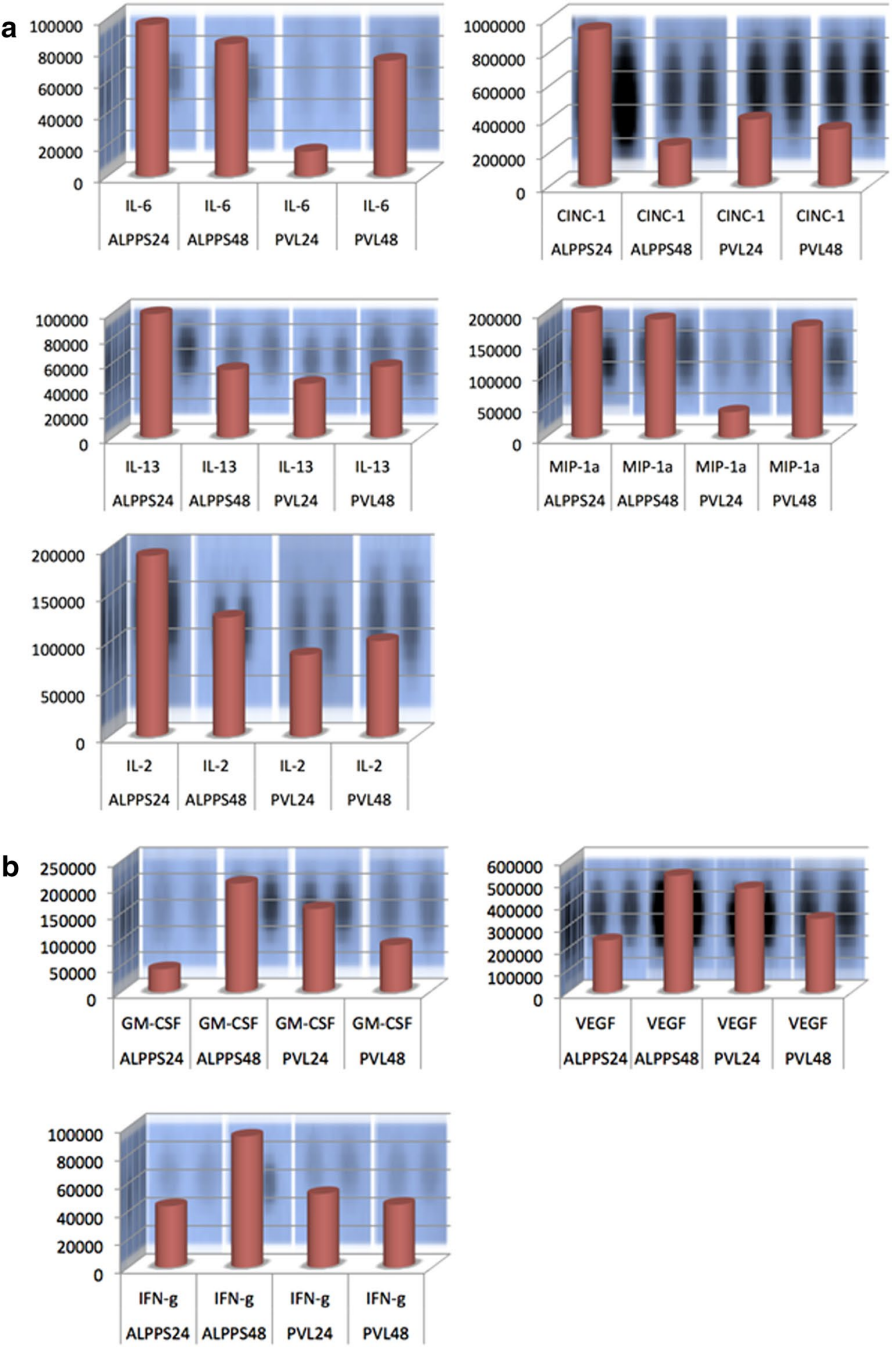
The t*op* 5 (**a**) *panels* show the relative expression of cytokines/chemokines in the liver tissue whose expression were more than twice in the ALPPS group than in the PVL group at early hours of liver regeneration (24 h). Whereas *bottom* three *panels* (**b**) show the expression of proteins whose expression was more than twice at 48 h time pint in the ALPPS group than in the PVL group

To evaluate the possible mechanism of early regenerative response in the ALPPS group, next we looked at the expression of a battery of cytokines/chemokines and growth factors at the tissue level. We used a proteome array kit to examine the expression profile of 29 cytokines/cytokines in liver tissue samples. The list of the proteins with their mean OD values is shown in Additional file 3: Table S1. We have divided them into early responsive or late responsive proteins depending on whether the expression of the proteins in the ALPPS group was higher at 24 or 48 h time points. Figure 6 shows the mean optical density (OD) values of the proteins whose expressions in the ALPPS group were more than twice than the expression in the PVL group. Among all of the cytokines, the cytokine-induced neutrophil chemoattractant -1 (CINC-1) had the strongest expression whereas the largest difference in expression between the ALPPS and PVL group was in interleukin-6 (IL-6) expression with more than 6-fold higher expression in the ALPPS group than in the PVL group at 24 h. Besides IL-6 and CINC-1, (interleukin-2)IL-2, (interleukin-13) IL-13 and macrophage inflammatory protein 1alpha (MIP-1a) expressions were higher(>2 fold) in the ALPPS group at 24 h time point than in the PVL group (Fig. 6a). In contrast, only granulocyte macrophage colony stimulating factor (GM-CSF) had more than 2-fold expression in the PVL group than in ALPPS group at 24 h. Also we noticed expression of granulocyte macrophage colony stimulating factor (GM-CSF), vascular endothelial growth factor (VEGF) and interferon gamma (IFN-g) with late peaks at 48 h in the ALPPS group when compared with the PVL group (Fig. 6b).

## Discussion

The ALPPS concept of inducing fast-track liver regeneration might be one of the finest surgical marvels in the field of liver surgery in recent decades. In this study, we have reproduced the clinical concept of ALPPS in a rat model and, as far as we are aware, this is the first full report of rat ALLPS model. We have convincingly showed that the ALPPS procedure steers robust liver regeneration at a much faster rate than the PVL alone, however, the PVL itself produced comparable amount of liver regeneration in the long run at 1 week postoperatively.

During the ALPPS procedure, usually the segments 2 and 3 are spared as the FRL, which constitute about 20–25 % of the whole human liver. We choose the rat model as the rats are widely used for the liver regenerative studies and easily available for the research purposes, however, in rats the liver is lobulated except the median lobes which constitutes about 38.17 ± 1.45 % of the whole rat liver with approximately 25 % belonging to the right median lobe (RML) and 14 % to the left median lobe (LML). Therefore, to reciprocate the clinical ALPPS model in terms of volume of FRL, we ligated portal branches to all liver lobes except the right mead liver lobe which constitute approximately 25 % of the rat liver. We used the median lobe for the liver parenchymal partitioning along a line just right to the falciform ligament. We noticed significant regenerative drive of the FRL in the ALPPS group at 24 h time point whereas the regeneration kicked off in the PVL group at a later stage at 48 h. In the ALPPS group, the FRLV/BW ratio was higher at all points when compared with the PVL group except at 1 W time when the FRLV/BW ratio became almost same in both groups.

While this article was in preparation, we noticed a report describing the ALPPS model in mice which used the left median lobe (13 % of the whole liver) as the FRL and also included an additional step of left lateral lobectomy (30 % hepatectomy) in addition to the ALPPS procedure [9]. The authors of this article concluded a significant increase in the FRLV/BW ratio in the ALPPS group when compared with the PVL group and our results concur with their findings, however, only at the early stage of the liver regeneration. In our model, the FRLV/BW ratio was marginally higher in the PVL group when compared with the ALPPS group at 1 week time point whereas in their study the volume of the regenerating lobe was higher in the ALPPS group than in the PVL group (p = 0.06). This could be attributable to the design of their model, which comprised of an additional partial hepatectomy (30 %) and ALPPS rather than the ALPPS only model which might had an additional advantage for the ALPPS group but not for the PVL group. In rodents, the left portal vein branch divides distally into two branches; the upper one supplies the left median lobe and the lower branch supplies the LLL. As the LLL branch is embedded into the hilum of the LLL parenchyma, therefore, it remains quite challenging to selectively ligate this branch without interfering the blood supply to the LML and LLL. In a pilot study, we tried to selectively ligate the LLL portal branch to create a left-sided ALPPS model without removing the LLL, however, the results were inconsistent (data not shown). From this viewpoint, we prefer the right-sided ALPPS model (right median lobe as the FRL) for further evaluation. Also when we were about to submit this article, we noticed a brief report on right sided rat ALPPS model mentioning significant increase in liver regeneration of the FRL at day 3, 7 and 14 but lacking any comparison with the PVL group [10]. AS far as we aware, this is the first study showing the liver images of the ALPPS regenerated lobes in an animal model.

Liver regeneration is a complex procedure involving a concerted activity of different kinds of cells including the resident liver cells and infiltrating inflammatory cells which is triggered by shear stress, cytokines and growth factors [11]. The doctrine behind the ALPPS was to prevent the neocollateral formation across the ligated and non-ligated liver lobes by adding the liver parenchymal partitioning step to the PVL. It seems that the portal vein ligation/embolization alone often fails to induce enough contralateral hypertrophy because of distal portoportal collateral formation [12] and also in experimental study it has been shown that proximal portal vein occlusion causes progressive portal cavernoma and reentry of portal blood from the non-occluded lobe to the occluded lobe [13]. In this study, we noticed significant increase in the portal pressure in the ALPPS group compared with the PVL group, which might have increased the shear stress and stimulated early regeneration in the ALPPS group. It is worth to mention that Shindoh et al. [14] reported a unique way to perform the PVE which also includes the segment four producing an equivalent FRL hypertrophy compared to the ALPPS, however, at a relatively slower rate which the authors consider as an advantage over ALPPS from the perspective of detection of occult metastatic focus before the 2nd stage operation and thus the unnecessary 2nd stage operation could be avoided. A comparable liver regeneration at one-week time point in both the ALPPS and PVL groups could be explained by the unique PVL approach we did in this study which includes part of the right median lobe and may represent the segment four in human. This needs further evaluation in an experimental set up.

In addition to putting a physical barrier to prevent the formation of portoportal collateral formation, liver partitioning along with the PVL induced cellular apoptosis and a sterile inflammatory reaction in the liver followed by cytokine surge; which in turn helped in accelerated liver regeneration. In our study, we noticed early increase in inflammatory cell infiltration in the liver tissue in the ALPPS group. Similarly, Tanaka et al. [15] mentioned almost twice the number of hepatic kupffer cells infiltration following PVE + PH than in the PH only group which also provided better protection following endotoxin challenge in rats. We examined an array of cytokines in the liver tissue and, according to the kinetics of them we propose a concerted involvement of hepatic parenchymal cells, resident non-parenchymal cells and infiltrated inflammatory cells in the augmented ALPPS liver hypertrophy. It seems that the a concerted involvement of immunomodulatory cells including the hepatic stellate cells, kupffer cells, macrophages and bone marrow derived cells play pivotal roles in the liver regeneration. Among the 29 examined cytokines, CINC-1 had the strongest and early significant expression in the ALPPS liver when compared with the PVL liver, supporting a crucial role of CINC-1 in the ALPPS associated regeneration. Interestingly, Kaibori et al. [16] noticed that CINC-1 induced by hepatocyte growth factor (HGF) produced enhanced proliferative and angiogenic activities through NFkB activation in the liver. Besides CINC-1, we also noticed early significant expression of IL-6, MIP-1a, IL-2 and IL-13 in the ALPPS group which might have played significant role in the ALPPS liver regeneration. The increased IL-6 level concords with the mice ALPPS study as well, the authors noticed a significant increase in plasma IL-6 in the ALPPS group compared with the PVL group. It is particularly worth to mention that early activation of IL-6/STAT3 pathway in macrophages is essential for the production of chemokines such as MIP-a, which in turn might enhance the migration of bone marrow derived MSCs to the liver during the restitution of liver mass [17]. In fact, we noticed a significant increase in MIP-1a in ALPPS liver at 24 h when compared with the PVL group (Aure 6). These findings are in agreement with the differential expression pattern and kinetics of cytokines/growth factors in PH and PVL. Fujii et al. [18] showed that follistatin, a TGF-B/activin binding protein, accelerated the regeneration following PH, however, had no effect on regeneration induced by the PVL whereas Mueller et al. [19] noticed that the mRNA of cell proliferation marker histone2B significantly elevated at 24 h after PH but it was delayed at 48 h following PVL. Therefore, our results show differential expression of cell proliferation activating chemokines, specially the CINC-1-MIP1 –IL-6 axis, during the early regenerative phase between the ALPPS and PVL group, further studies are necessary to modulate the expression of particular growth factor such as CINC-1 during the ALPPS liver regeneration model in the set up of parenchymal liver disease to further augment liver regeneration.

Liver has a unique capability to regenerate and the actual mechanism(s) of the regeneration still remains a dilemma. Studying the mechanism of regeneration in the ALPPS model would unlock the myth behind the extraordinary capability of the liver for regeneration, which would help in designing new therapeutic options for the regenerative drive in difficult set up such as chronic liver diseases. Although the ALPPS procedure is getting popularity among the surgeons, however, the procedure is still in it’s infancy needing further refinement for broader clinical application [20]. Following the ALPPS procedure, the percent increase in the FRLV ranges from as low as 21–200 % and, to date, the actual reason of this massive variation is not clear. This could be partially attributable to the presence of underlying parenchymal liver diseases such as metabolic syndrome, cirrhosis and neoadjuvant treatment related liver diseases such as sinusoidal obstruction syndrome. The effect of presence of these conditions on ALPPS regeneration may help to improve our understanding of ALPPS regeneration and would decrease the postoperative morbidity. Also other areas which will need attention to further increase the indication of ALPPS include the natural history of growth of micrometastasis in the FRL, role of the atrophic lobe on the regeneration of the FRLV, and, chemical, non-invasive or minimally invasive liver partitioning are among a few. We believe that our ALPPS model in rats would enable the researchers to address these unsolved issues related to the ALPPS in the future.

## Additional files

10.1186/s13022-015-0020-3 ALPPS liver with necrosis of the left median lobe and necrosis of the transection line are shown.
10.1186/s13022-015-0020-3 The mean portal pressure (mm of hg) is shown. Portal pressure was higher in the ALPPS group compared with the PVL group at most of the time points, however, it was significant only at 24 h time point.
10.1186/s13022-015-0020-3 The mean optical densities of cytokenis/chemokines.

## Abbreviation

ALPPS: associated liver partitioning and portal vein ligation for staged hepatectomy
FRLV: future remnant liver volume
AST: aspartate aminotransferase
ALT: alanine aminotransferase
CINC-1: cytokine-induced neutrophil chemoattractant -1 (CINC-1)
IL-6: interleukin-6 (IL-6)
PVL: portal vein ligation
IL-2: interleukin-2
IL-13: interleukin-13
MIP-1a: macrophage inflammatory protein 1alpha
GM-CSF: granulocyte macrophage colony stimulating factor
VEGF: vascular endothelial growth factor
IFN-g: interferon gamma
