# Supplementary figures and images for: A novel rat model of liver regeneration: possible role of cytokine induced neutrophil chemoattractant-1 in augmented liver regeneration
Source: Ann Surg Innov Res. 2015 Nov 2;9:11. doi: 10.1186/s13022-015-0020-3 (PMC4631081; doi:10.1186/s13022-015-0020-3)

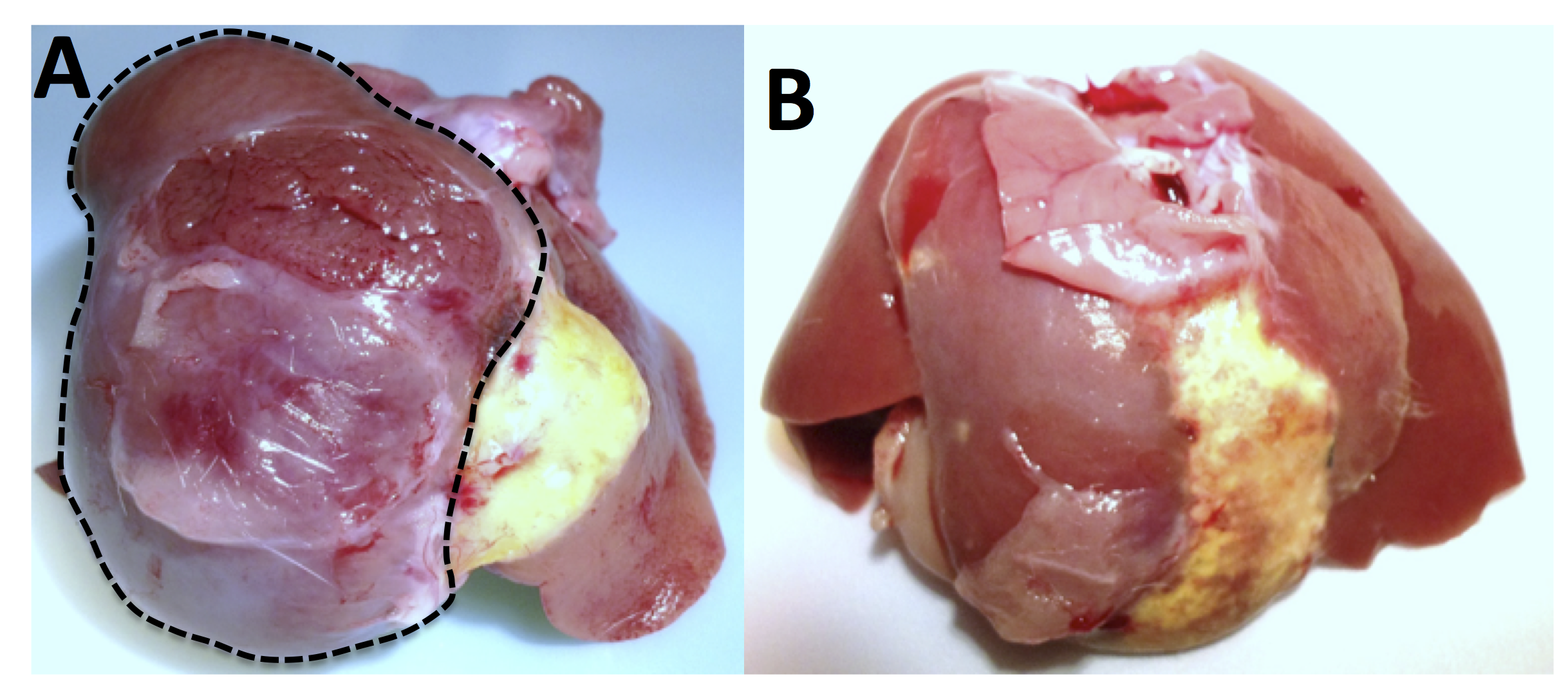

Supplement: Supplementary file 1 — 10.1186/s13022-015-0020-3 ALPPS liver with necrosis of the left median lobe and necrosis of the transection line are shown. [file 13022_2015_20_MOESM1_ESM.tiff]

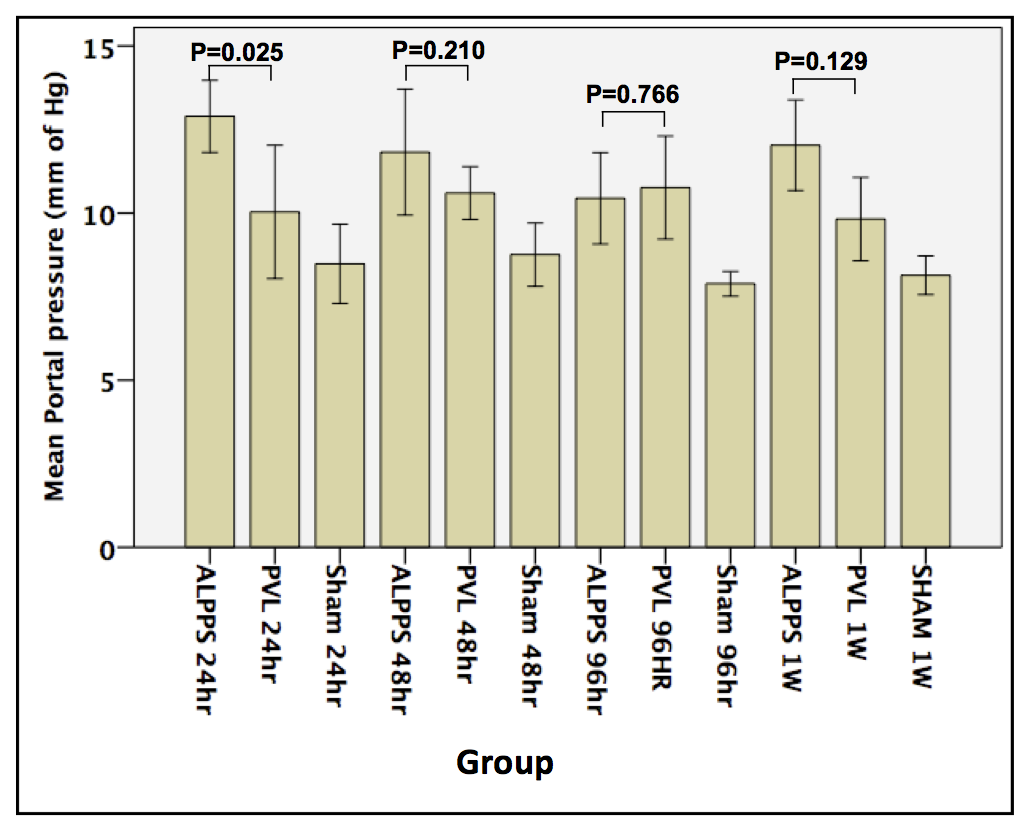

Supplement: Supplementary file 2 — 10.1186/s13022-015-0020-3 The mean portal pressure (mm of hg) is shown. Portal pressure was higher in the ALPPS group compared with the PVL group at most of the time points, however, it was significant only at 24 h time point. [file 13022_2015_20_MOESM2_ESM.tiff]

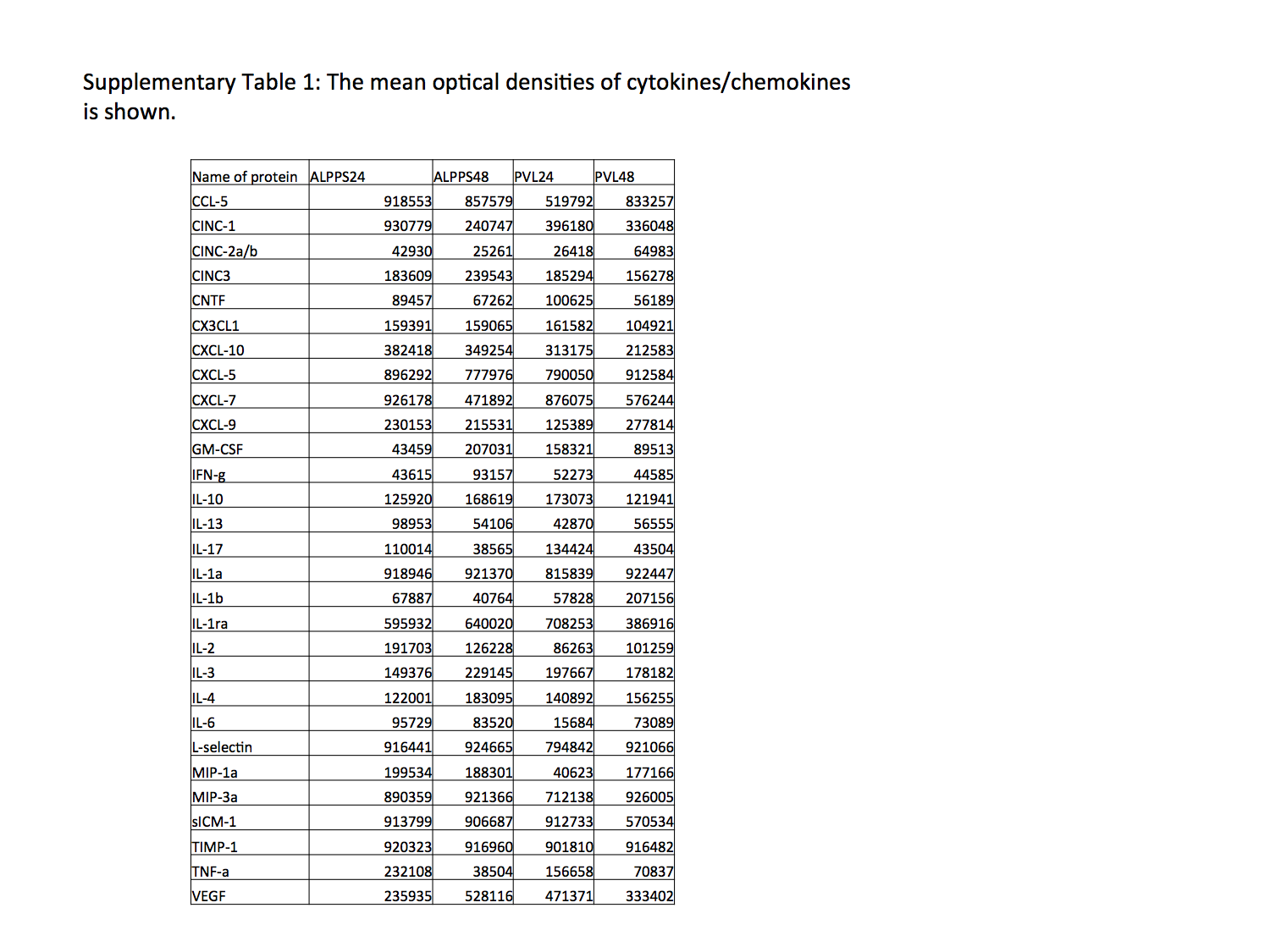

Supplement: Supplementary file 3 — 10.1186/s13022-015-0020-3 The mean optical densities of cytokenis/chemokines. [file 13022_2015_20_MOESM3_ESM.docx]
